# A Review of Environmental Risk Factors for African Swine Fever in European Wild Boar

**DOI:** 10.3390/ani11092692

**Published:** 2021-09-14

**Authors:** Hannes Bergmann, Katja Schulz, Franz J. Conraths, Carola Sauter-Louis

**Affiliations:** Friedrich-Loeffler-Institut, Federal Research Institute for Animal Health, Institute of Epidemiology, Südufer 10, 17493 Greifswald-Insel Riems, Germany; katja.schulz@fli.de (K.S.); franz.conraths@fli.de (F.J.C.); carola.sauter-louis@fli.de (C.S.-L.)

**Keywords:** African swine fever, risk factor, wild boar, epidemiology, disease control, surveillance, environment

## Abstract

**Simple Summary:**

African swine fever (ASF) is a viral haemorrhagic pig disease that continues to spread in Europe and severely damages pig production and economy, disrupts trade with pigs and porcine products and even has an impact on social welfare in affected areas. Wild boar and domestic pigs are both susceptible to infection with the ASF virus, which causes generalised haemorrhagic illness, fever and rapid death of most infected animals within a few days. ASF occurrence in wild boar dominates the spread and persistence of this disease in Europe and poses an imminent threat for spill-over infections with ASFV in domestic pig holdings. Wild boar represent an intelligent and adaptable wildlife host for ASF with an expansive distribution range in Europe and complex biology. Wild boar thus intricately link ASF with their habitat, making ASF inherently complicated and resource-hungry to control in the environment. This work reviews the currently known environmental risk factors for ASF in wild boar and specifically assesses the role that climate, land cover, human activity, wild boar and disease distribution play in the occurrence of ASF in wild boar. The reviewed risk information guides the implementation of ASF control measures in wild boar.

**Abstract:**

A detailed understanding of environmental risk factors for African swine fever (ASF) in wild boar will be not only essential for risk assessments but also for timely and spatially informed allocation of resources in order to manage wild boar-targeted ASF control measures efficiently. Here, we review currently known environmental risk factors that can influence the occurrence of ASF virus infection in wild boar when compared to disease occurrence in wild boar of a non-exposed reference scenario. Accordingly, the exposure of wild boar to environmental risk factors related to (1) climate, (2) land cover, (3) human activity, (4) wild boar and (5) ASF were evaluated. As key environmental risk factors in this review, increased ASF occurrence in wild boar was associated with seasonal patterns, forest coverage, presence of water, human presence, farming activities, wild boar density and ASF nearness. The review highlights inconsistencies in some of these risk factor associations with disease detection in space and time and may provide valuable insights for the investigation of ASF transmission dynamics. The examined risk information was applied to consider potential improvements of the ASF control strategy in wild boar regarding disease surveillance, hunting, wild boar carcass searches and ASF barrier implementation.

## 1. Introduction

African swine fever (ASF) has continuously spread in Europe in recent years. The current Eurasian epidemic originated in Georgia in 2007. From there, it first spread in the Caucasus region, then north and northwest, finally reaching central Europe, but also spreading east into Asia [1]. The causative agent of this disease, ASF virus (ASFV), is a large, complex and enveloped DNA virus that is characterised by its high environmental stability [2,3,4,5]. Especially at low temperatures, infectious ASFV can be recovered from pig tissues for several months, or even years, particularly from blood, muscle and skin tissues kept at −20 °C or 4 °C [6,7,8,9]. ASFV infects only pigs; it is harmless to humans. In European wild boar and domestic pigs, infection with ASFV usually causes severe generalised illness, leading to fever within a matter of days, bleedings and rapid death of most infected animals [5]. The presence of ASF in a region severely damages pig production and economy, disrupts trade in pigs and porcine products and may even affect social welfare [10,11]. Therefore, informed, risk-based decisions are not only needed to allocate limited resources for ASF control in affected regions but also to protect disease-free regions from new ASF introductions. Whilst no sufficiently protective ASF vaccine is currently available [11], the development and inclusion of vaccination strategies into future ASF control programs should be considered on risk-based principles [1,12,13], thus maximising the effectiveness of ASF vaccines that may become available in the future [14]. Taken together, these considerations highlight the need for ASF risk factor identification and mitigation [15].

For the identification of ASF risk factors, we must consider the current epidemiological situation, the predominant disease hosts, potential pathogen vectors or ‘vehicles’ that could relocate ASFV and the environmental backdrop of ASF disease control in Europe. With the exception of two resolved point incursions of ASF, one into the Czech Republic [16] and one into Belgium [17], disease spread has so far been very difficult to control in the current European epidemic.

**Figure 1:**
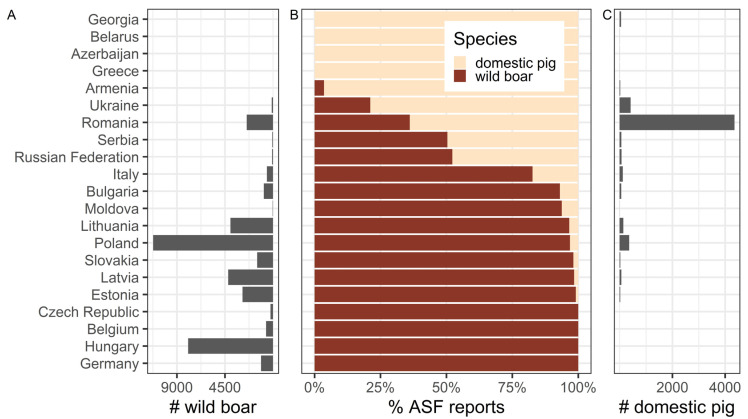
Horizontal bar graphs summarising the absolute number of ASF reports in wild boar (**A**), or domestic pigs (**C**), as well as the relative proportions of ASF, reports for each pig species (**B**) in the indicated countries from April 2007 to May 2021. The mean percentage of the horizontal bar graph in panel B is 62.95%. ASF—African swine fever; Source: OIE, ADIS; considered reports include the detection of ASFV genome (PCR) and humoral immune responses to ASFV (ELISA).

Since 2007, approximately 47,000 wild boar cases and domestic pig outbreaks of ASF have been reported overall in Europe [18]. The vast majority of these notifications, at over 86%, were made for ASF in wild boar. Less than 14% of the reported events concerned ASF in domestic pigs, indicating that wild boar currently represent the predominant host for ASF in Europe (Figure 1).

**Figure 2:**
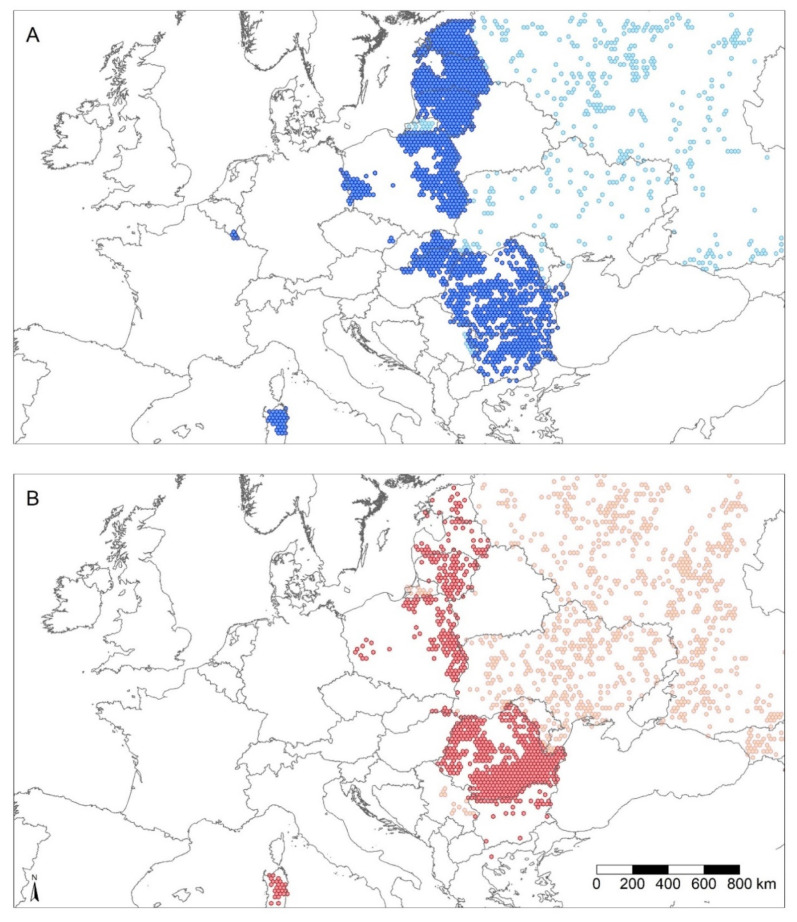
Area estimate maps of European countries affected by ASF in wild boar and domestic pigs. The maps show an estimate of the spatial extent of ASF in Europe, comparing ASF reports from April 2007 to May 2021 in wild boar ((**A**), blue), with reports in domestic pigs ((**B**), red) mapped to a 20 km hexagonal grid across the shown area. One 20 × 20 km grid cell covers an area of 346.41 km^2^. Grid cell regions with ASF reports in countries of the EU are marked by dark blue (wild boar, (**A**)) or dark red colour (domestic pig, (**B**)), whereas grid cell regions with ASF reports outside of EU countries are indicated by light blue (**A**) or light red colour (**B**), respectively. Clear areas of the map with no grid cells shown did not report ASF. The total number of ASF affected grid cells during the mapped time segment in all EU countries (dark cells only) was 1772 cells for ASF reports in wild boar ((**A**), total area of approximately 613,839 km^2^) and 892 cells for ASF reports in domestic pigs ((**B**), total area of approximately 308,998 km^2^). EU—European Union; ASF—African swine fever; Source: OIE, ADIS; Considered reports include the detection of ASFV genome (PCR) and humoral immune responses to ASFV (ELISA).

This conclusion also appears to hold true if the ASF-affected areas in Europe are compared on the basis of individual ASF report distribution. If a 5 km buffer is applied to each ASF report as a simplified proxy for the affected area around each notification and the resulting area is subsequently merged for all reported cases and outbreaks, ASF reports in wild boar relate to a 2.51-fold larger total area (350,193 km^2^) than ASF reports in domestic pigs (139,391 km^2^). A 5 km buffer was chosen as a crude estimate of the area that could be affected by ASF in the vicinity of ASF reports, potentially through animal or human-mediated movements. However, this type of comparison might be biased across all of Europe due to differences in surveillance and ASF reporting among the countries that were considered, as shown in Figure 1. Furthermore, individual ASF reports in domestic pigs generally refer to farm outbreaks with multiple pigs involved, whereas reports in wild boar usually denominate only a single animal. We, therefore, disengaged spatial ASF distribution from individual reports by mapping ASF occurrence to 20 km hexagonal grid cells and only included countries of the European Union, for which similar surveillance and reporting procedures could be assumed (Figure 2). Consistent with the report-based estimation, the grid-based approach showed that ASF in wild boar (613,839 km^2^) affected an area almost twice as large as ASF in domestic pigs (308,998 km^2^) within the European Union (Figure 2). Additionally, on a per-country basis, the disease in wild boar predominates in most ASF-affected countries in all of Europe (12 out of 21) and the European Union (11 out of 13) (Figure 1b).

The detection of ASF in places that were far away from previously known outbreak areas highlights the importance of human activities as a factor associated with ASF spread [19,20,21,22]. Notably, some of these long-range, likely human-mediated ASF incursions that affected the Czech Republic [16], Belgium [17] and West Poland [23,24] occurred in wild boar. Once ASF has been introduced into a previously ASF-free area, the disease can spread among wild boar through currently recognised ASF transmission pathways. These include direct pig-to-pig interaction and indirect contact, e.g., through carcasses of wild boar that died of the disease and environmental contamination [1,20,21,25]. Particularly the carcass-mediated transmission pathway seems to represent a characteristic mechanism of ASF spread in the current European context [2,3,7,25,26]. The introduction of ASF into a naïve wild boar population initially results in disease-mediated deaths and increases the environmental density of wild boar carcasses [27]. The environmental persistence of ASFV in the decomposing carcass is understood as a source of infection for wild boar [7,26]. The association of ASF with wild boar and environmental factors in the current European scenario and the resulting occurrence of ASF in wild boar habitats has led to the description of a ‘wild boar habitat’ ASF transmission cycle [28,29]. It thus appears fair to conclude that wild boar play a major role in ASF spread and persistence in Europe. Complex wild boar biology and environmental factors that influence the wild boar habitat should therefore be a major focus for ASF control efforts [30].

From this perspective, the relevance of wild boar biology and ASFV-persistence in wild boar carcasses highlight environmental risk factors as a useful category of risk factors to focus on [30]. ASF does not occur in a vacuum, and it is therefore helpful to consider the concept of the epidemiological triad (pathogen, host, environment) to define environmental risk factors [31,32].

The epidemiological triad highlights appropriate categories that need to be considered as elements of disease causation. Thus, for the purpose of this review, an environmental ASF risk factor for wild boar was defined as follows: any factor that occurs in the environment and exposes wild boar populations, which then become infected with ASFV, whereas a comparator wild boar population that has not been exposed to the factor as a suitable reference scenario does not become infected.

It is the objective of this work to review the current literature reports about environmental risk factors associated with ASFV infection in European wild boar, but not domestic pigs, and to review factors, which are, as much as possible, in line with the provided definition. Therefore, the scope of this paper is limited to review only studies that compare ASFV infected and non-infected wild boars for their exposure to environmental risk factors. The selection of relevant publications in accordance with the objective for this narrative review was identified based on systematic literature review principles (manuscript in preparation). The environmental-type of ASF risk factors in relation to wild boar populations are considered especially relevant in the European context. A thorough understanding of environmental ASF risk factors in wild boar is expected to help explain ASF transmission dynamics, risks of ASF incursion and spread, but also to critically inform resource allocation and strategy development for ASF surveillance and control efforts in wild boar.

## 2. Environmental ASF Risk Factors

**Figure 3:**
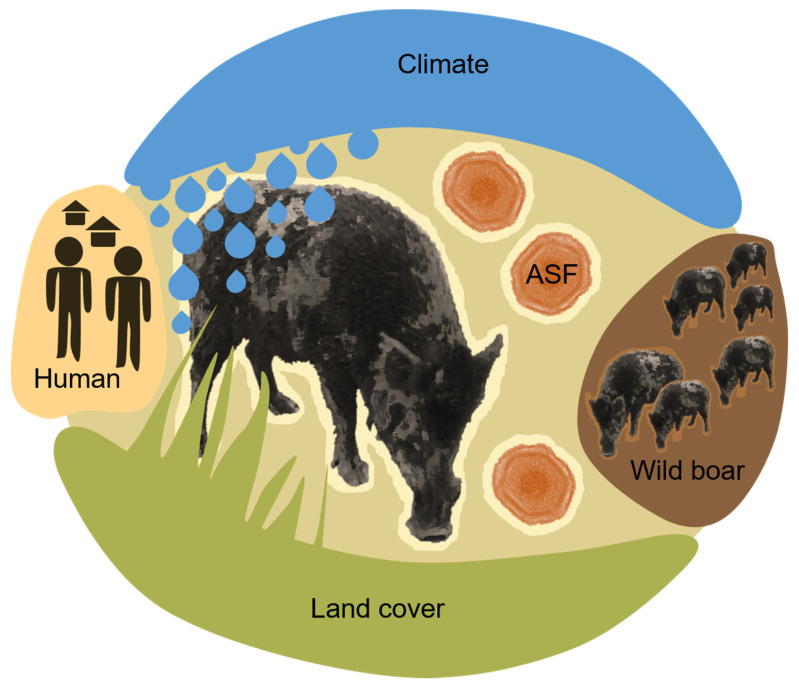
Environmental ASF risk factors for ASF in wild boar. Schematic drawing illustrating the reviewed environmental risk factor elements for ASF in wild boar. ASF—African swine fever.

When considering the environment of ASFV infected wild boar in Europe, several elements of biotic and abiotic factors come to mind that influence or occur in the environment and could be investigated in detail for their association with ASF. As illustrated in Figure 3, these elements non-exhaustively include:

**Climate factors**, such as temperature, precipitation, humidity, wind, cloud coverage, ultra-violet light conditions, climate changes or season;
**Land cover and geomorphology factors**, such as vegetation-type, coverage, distribution pattern, altitude, soil type and water availability or type;
**Human activity factors**, such as human population density, traffic, pollution, artificial structures, housing, roads, farm density, livestock density as well as human outdoor activity types and levels;
**Wild boar host-related factors**, such as wild boar presence in terms of density, distribution or measurable effects as a result of their activity (e.g., crop damage);
**ASF disease factors**, such as disease presence, disease type (e.g., a high proportion of ASFV seropositive wild boar present), distribution, distance in space and time from susceptible animals and the viral load, infectious pressure or contamination level.

## 3. Climate

Many climate parameters are readily measurable and have a pervasive impact on life. Therefore, understanding the potential association of ASFV infection in wild boar with any climate factor would not only provide a well-defined determinant of disease and guide the mechanistic investigation but also offer an easily applicable indicator of disease risk. Investigation of climate factors over longer periods could reveal the association of ASF with climate change. Climate indicators could then be used to allocate or adapt disease control efforts in space and time.

### 3.1. Seasonality

Whilst detailed weather or climate parameters, such as temperature or rain, might be quite variable over space and time, the broad change in climate that occurs through seasonal fluctuations each year appears to be a robust indicator that could be particularly suitable for examination of association with far-spreading trans-boundary diseases, such as ASF. Along with seasonal change, the behaviour of wild boar undergoes distinct changes. Parameters such as higher temperatures, associated insect and scavenger activity or ultra-violet radiation change and may influence the degradation rate of ASFV in the decomposing carcass, and also the activities of humans interacting with wild boar habitats may change from season to season [9,33,34]. While the interplay and relevance of these factors are unclear, it would be reasonable to expect a seasonal pattern of ASF occurrence.

To examine seasonality as an environmental risk factor, ASFV infections in wild boar exposed to a specific season would have to be measured and compared to the infections detected in wild boar that are not exposed to the season under examination. Samples collected in Poland between 2014 and 2016 from found-dead or hunted wild boar were tested for the presence of the ASFV genome. Temporal analysis consistently identified a seasonal peak of ASFV infected wild boar during summer in several studies [21,35,36]. By contrast, similar studies analysing data from 2014 to 2017 in Lithuania identified a seasonal peak of ASFV genome-positive samples from found-dead wild boar during winter, whereas viral genome-positive samples from hunted wild boar rather peaked during summer to autumn [37]. These findings were confirmed later by further data from Lithuania through to 2018, which also identified a seasonal peak of ASFV-infected wild boar among found-dead animals in winter and a peak of viral genome-positive samples among hunted wild boar in autumn [38].

An overarching analysis of similar data from several European countries by the European Food and Safety Organisation (EFSA) was based on sample collections from found-dead or hunted wild boar in the Baltic States, Poland, Czech Republic, Hungary and Belgium between 2014 and 2019 [9,19,22,39]. Early analyses, looking at data up to 2018, found seasonal peaks of ASFV genome-positive wild boar (found-dead animals) in the summer for Poland, Latvia and Estonia, but less pronounced for Lithuania, and in winter for Poland, Lithuania and Latvia, but not for Estonia. By comparison, a seasonal peak for viral genome detection among hunted wild boars was noticeable for late summer and autumn in all Baltic States but not in Poland. Statistical testing confirmed the existence of seasonal variation in the detection probability of ASFV infected wild boar; however, a specific peak season could not be assigned across all examined countries [9]. An update of the same analysis for detection of ASFV genome in found-dead wild boar included data from more European countries and expanded the study period up to August 2019 [22]. These analyses confirmed summer peaks in Latvia and Estonia and, to a lesser degree, in Lithuania and Poland, winter peaks for all Baltic States, Poland and the Czech Republic, whereas for Hungary and Belgium, peaks in spring were observed [22]. The proportion of ASFV genome detections among all hunted and tested wild boar was again higher in late summer and autumn in the Baltic States, whereas in Poland, ASF detection among hunted wild boar peaked in summer and winter. For the Czech Republic, Hungary and Belgium, seasonal variation in hunted wild boar were unclear, probably due to smaller affected areas and the shorter periods during which ASF was present in these regions [22]. Seasonal differences in the probability to detect the ASFV genome in either found-dead or hunted wild boar were again statistically confirmed for all examined countries [22]. In conclusion, it appeared that seasonal patterns for the occurrence of ASF in wild boar exist. Nevertheless, these patterns were inconsistent in space and time.

### 3.2. Precipitation and Temperature

Specific climate factors, such as temperature or precipitation, could potentially influence the occurrence of ASF in wild boar, and observations in association with these parameters might help to explain the seasonality of ASF occurrence. Liang et al. modelled the global ASF occurrence and examined the contribution of bioclimatic variables in confirming ASF occurrence based on the model prediction [40]. The underlying model was developed with global ASF outbreak data that did not differentiate whether the disease occurred in wild boar or domestic pigs. It was found that meteorological information was strongly correlated with the overall global ASF occurrence. Particularly, precipitation measures strongly contributed to confirming ASF occurrence, including precipitation in the driest month, precipitation in the coldest quarter and precipitation in the driest and wettest quarter [40]. Temperature-related bioclimatic variables also contributed to the modelled ASF occurrence predictions. Dominant variables included the minimal temperature of the coldest month and the mean temperature of the coldest quarter [40]. These findings indicate that precipitation, particularly during potential low rainfall seasons, might represent factors that influence ASF occurrence globally. Likewise, temperatures during cold seasons appear to be relevant indicators for global ASF occurrence. By contrast, examination of the ASF occurrence in Estonian wild boar from 2014 to 2019 and testing possible associations with temperature or precipitation measures did not reveal any significant contribution of either yearly average minimum temperatures or average yearly snow depth in a hierarchical Bayesian approach modelling the occurrence of ASF [9,22]. Investigations of found-dead wild boar on a much smaller spatial scale (hundreds of meters) in the Czech Republic suggested that increases in mean air temperature reduced the average distance of ASFV infected wild boar occurrence in relation to water sources, which would not be detectable at a regional or global level [16].

In summary, ASF occurrence in European wild boar follows inconsistent seasonal patterns and meteorological parameters seem to be able to predict the global occurrence of ASF coarsely. However, it is unclear whether the disease is directly influenced by climate factors, and if so, which factors matter. It is also possible that climate factors have rather indirect effects on ASF by modifying wild boar behaviour, human behaviour, influencing plant growth or the persistence of ASFV in the environment.

## 4. Land Cover

The presence and distribution pattern of various land cover types defines the direct interface of ASF on the surface of the earth. Wild boar as ASF hosts depend on minimal land cover requirements to support sustainable populations. Thus, land cover greatly influences wild boar habitat quality, distribution ranges, and ultimately, the spatial distribution of ASF occurrence [41,42]. Moreover, land cover types may also determine the persistence of wild boar carcass material and associated ASFV in the environment by providing protection in the shade and thus minimising decay of infectivity in the sun or supporting scavenger biodiversity and insect activity [33]. Finally, land cover may also influence the accessibility of areas to ASF surveillance in wild boar, so that a land cover-mediated delay in disease detection might hold up implementation of control measures and potentially facilitate the establishment of ASF in inaccessible areas. Therefore, identification of land cover types and patterns that associate with the occurrence of ASF seems valuable for managing disease control measures in ASF-affected regions, for example, through guiding fence building or wild boar carcass search activities.

### 4.1. Forest

From a perspective of habitat suitability, any land cover type that provides the basic requirements of wild boar habitats regarding shelter, water and food would be important to consider in the context of environmental ASF risk factors [41,42,43]. Some forest types, such as broad-leafed nut-bearing trees, provide food and shelter. Forest growth supporting water abundance is a prerequisite for forest coverage in the first place. It is probably sufficient to sustain wild boar populations, thus highlighting forest coverage with appropriate tree species as a key land cover type to satisfy the minimal requirements for a wild boar habitat [41]. Consistent with this view, forest coverage at a regional scale was found to associate with the occurrence of ASF in wild boar across distinct geographical regions in Europe, including the Baltic States [39] and Italy [44], indicating that the probability of detecting ASF in wild boar is greater in regions with large forest-covered areas.

At finer spatial scales, which should be more representative of wild boar home-range structures, observations similar to the regional forest studies were made in Poland. When the proportion of forest coverage surrounding a tested wild boar carcass within a 2 km radius was examined, it was found that the probability of detecting the ASFV genome in a wild boar carcass increased with increasing proportions of forest coverage surrounding the animal [36]. Small, spatially scaled studies, conducted in the Czech Republic in the area that was affected by ASF from 2017 to 2018, provided additional information about the relative influence of forest coverage on detecting ASF in wild boar [16]. Whilst a homogeneous search effort throughout the relatively small ASF-affected area can be assumed, over 70% of all carcasses in this area were found in forests. However, by comparison to other land cover types, the odds of detecting ASFV infected wild boar were actually greater in the meadow (OR 1.98) and field (OR 1.61) areas but lower in wetlands (OR 0.87), although fewer carcasses were found in these types of land cover overall [16]. In the same study, it was also found that the probability of detecting ASFV infected wild boar increased in juvenile forest stands aged 40 years or younger and at greater distances from forest edges, although the majority (over 80%) of carcasses were found within 200 m of the forest edge [16]. Notably, almost no wild boar carcasses were found more than 500 m away from forest edges, highlighting forest edges and associated land cover transition zones as key areas for ASFV-infected wild boar detection and thus for surveillance [16]. Overall, logistic regression modelling conducted to explain spatial detection patterns of ASFV-positive wild boar carcasses in the Czech Republic outbreak area revealed that (younger) forest stand age and the ratio of broad-leafed forest trees (their increased presence) were significant predictors of finding ASFV infected wild boar carcasses [16].

### 4.2. Water and Meadows

Whilst forest land cover potentially satisfies wild boar habitat needs, the explicit presence of surface water appears to be another key land cover factor for ASF occurrence. The occurrence of ASF in wild boar in the Baltic States and Poland from 2014 to 2016 was examined for association with surface water by using a classification tree model [39]. During the examined period, it was found that for the Baltic States, but not Poland, the probability of detecting at least one ASF-positive wild boar case was associated with the proportion of maritime wetlands, inland wetlands (inland marshes and peat bogs) and water bodies, which included watercourses, water bodies, coastal lagoons and estuaries [39]. Investigations of the detection of wild boar carcasses in relation to their distance to surface water sources in the Czech Republic revealed that 59.6% ASFV-positive and 76.2% ASFV-negative were found within 100 m of water. Almost all wild boar carcasses were found within 500 m of water sources, albeit these measures appeared to be air temperature-dependent [16]. Characteristics of meadow environments were also examined in the same study, suggesting that carcasses of ASFV-positive wild boar were more likely to be found in meadows with herb layer heights above 100 cm, whilst about 90% of all carcasses were detected in herb layers heights below 120 cm [16].

### 4.3. Wild Boar Habitat Quality

Individual land cover parameters might contribute to the probability of ASF occurrence in wild boar by supporting wild boar habitat requirements. Comprehensive modelling of the quality of wild boar habitats includes a range of land cover types and is a more direct way of examining the role of wild boar suitable land cover patterns in permitting ASF occurrence in wild boar [41,42]. Such an approach was taken by EFSA to analyse ASFV-genome detection data of wild boar found-dead in Estonia using a Bayesian hierarchical model. The analysis was repeated sequentially over several years [9,19,22]. It was found that average regional scores for wild boar habitat quality contributed only significantly to explaining ASF occurrence patterns in wild boar when ASF data from 2014 to 2017 were analysed. The analysis of Estonian ASF report data during this time segment suggested that the odds of detecting ASFV-infected wild boar increased by 0.74 for each unit increase in the habitat quality score [19]. Interestingly, the contribution of the wild boar habitat quality to explain ASF occurrence in wild boar in Estonia was not statistically significant when additional data from 2018 [9] and 2019 [22] were included in the analysis.

In summary, land cover types, such as forest and water, affect the occurrence of ASF in wild boar. It is unclear whether these effects are mediated through influences on wild boar habitat quality, ASFV persistence in the environment, local ASF surveillance and control efforts, a combination of these factors or potential interactions with further unknown parameters. An additional study is needed to examine the role of land cover factors in the occurrence of ASF in wild boar.

## 5. Human Activity

Human activities influencing the environment of ASFV infected wild boar are manifold. Human activity can directly cause the sudden spread of ASF over long distances [17,22,23,24], impact the environment, climate, land cover and consequently, wild boar habitats due to human presence, hunting or farming activities [9,45]. While climate and land cover factors are difficult to control and rather guide ASF management in space and time, it may be possible to regulate some types of human activity in the environment to help control ASF in wild boar. Hence, it may be useful to identify human activities in the environment that associate with the occurrence of ASF in wild boar.

### 5.1. Human Presence and Environmental Impacts

The simple presence of humans in numbers could be measured through population density. The influence of the human population on ASF in wild boar in a particular region was examined using classification tree models in the Baltic States from 2014 to 2016 [39]. During these years, an increasing human population density was associated with increased ASF occurrence in wild boar in Estonia and Lithuania, but not in Latvia.

While population density directly measures the presence of humans, additional factors can be considered to estimate the environmental impact of human presence, including urban coverage and the abundance of roads as indicators of transportation and residential development. Consistent with the effects found for human population density, the number of settlements was associated with the occurrence of ASF in wild boar in all Baltic States during 2014 to 2016 in the classification tree model [39]. However, these associations of population density and human settlement density/km^2^ as risk factors were lost if a Bayesian hierarchical model was used instead and information on ASF-positive and -negative wild boar from Estonia for 2017 [19], 2018 [9] or 2019 were analysed [22]. Similar findings were made in Poland, where no association of built-up areas with ASF in wild boar was found [36], and the Czech Republic, where the distance to settlements had no measurable effect on finding ASFV-infected wild boar carcasses when compared to non-infected carcasses [16].

The number of roads, road length and road density was found to have a variable influence on the occurrence of ASF in wild boar. From 2014 to 2016, an increasing abundance of roads was associated with disease occurrence in Latvia and Lithuania, but not in Estonia [39] and Poland [36]. The analysis of road abundance effects on the detection of ASF in wild boar in Estonia from 2014 to 2017 identified the regional road length as a risk factor that increased the odds of disease detection [19]. A similar analysis of Estonian data up to 2018 found the opposite effect, i.e., increasing road density reduced the odds of detecting ASF in wild boar [9], and analysis of Estonian data up to 2019 found no significant effect exerted by road density on disease occurrence [22]. By comparison, in the Czech Republic, ASFV-positive wild boar carcasses tended to be further away from roads (about 300 m) than ASFV-negative carcasses (about 100 m) [16], also suggesting a dispersing effect of roads on the probability of detecting ASFV-infected wild boar. Dead wild boar found in close proximity to roads could also be a result of road traffic accidents [46]. Experiences in Estonia and Latvia have shown that road traffic-associated wild boar deaths are not frequently examined for ASF and that the prevalence of ASF in wild boar that died by traffic accidents appears to be low [46]. These experiences suggest that road traffic accidents could contribute to increasing the occurrence of ASFV-negative wild boar carcasses in close proximity to roads slightly.

Renewable energy production and the generation of municipal waste represent interesting examples of outcomes of human activities in the environment, whereby particularly waste production is an obvious parameter in the context of ASF in wild boar. It appears plausible that increased waste production also increases ASF spill-over chances through littering by making ASFV contaminated pig products accessible to susceptible wild boar populations. From 2015 to 2018, Loi et al. examined the influence of waste production and energy production from renewable resources in Sardinian municipalities on ASF occurrence in wild boar. It was indeed found that municipalities with increased waste production observed more ASF cases in wild boar [44]. The production of energy from renewable sources had a protective effect and was associated with fewer observations of ASF infections in wild boar [44]. Whilst a relationship of waste production with ASF is conceivable, it is unclear how energy production relates to ASF occurrence in wild boar.

### 5.2. Hunting

Hunting and targeted wildlife management represent environmental human activities that directly affect wild boar. Hunting-related activities are thus expected to have a measurable effect on ASF in wild boar. However, repeated examination of the occurrence of ASF in wild boar and its association with various hunting-related activities in Estonia from 2014 to 2019 did not support this assumption [9,22]. Hunting and wildlife management associated parameters, including the density of hunters, the density of hunting dogs, the density of feeding or baiting places and the density of hunted wild boar, were not found to be associated with ASF detection in wild boar [9,22]. The reasons for the lack of association in this study are not known. It is possible that any wildlife management-related effects on wild boar populations and disease occurrence are so small when compared with ASF-related effects [27] that they are non-detectable in the examined data [9,22].

### 5.3. Farming

Farming likely influences the environment of ASFV-infected wild boar through most agricultural activities, which include crop production; effects of pig husbandry, such as manure management, pest control, feed storage, cadaver management, keeping pigs outdoor or in backyards, pig transportation; and travel and other activities of farm workers. Whilst many of these effects on ASF occurrence in wild boar may be difficult to measure, available information about the presence of farms, farmed livestock and farm types could be examined instead. In the Baltic States, the number of pig farms and the number of domestic pigs were indicators of the regional ASF occurrence in wild boar in Estonia and Latvia, but not in Lithuania, whereas the number of small pig farms increased the probability of disease occurrence in Latvia only [39]. Later in the ASF epidemic, the number of pig farms was identified as a risk factor for increasing the odds of detecting ASFV-infected carcasses when the ASF test results for wild boar carcasses from Estonia were analysed up to 2017 [19]. Similarly, data analysis from Estonia up to 2018 suggested the density of domestic pigs as a risk factor for the occurrence of ASF in wild boar [9]. Data ranging up to 2019 indicated that the density of pigs in small holdings keeping up to 10 pigs was a risk factor for ASF in wild boar [22].

In conclusion, human activities in the environment of ASFV-infected wild boar influence the epidemiology of the disease. Environmental risk factors for ASF related to the presence of humans and to farming were inconsistently identified among European countries over time, indicating that either disease dynamics or the influence of the examined risk factors may change in time and space, possibly in accordance with the local stage of the ASF epidemic. Hunting activities were not found to influence the occurrence of ASF in wild boar.

## 6. Wild Boar

Environmental risk factors for ASF in wild boar may consider the overall abundance or occurrence pattern of these animals in space and time, as well as any signs of their presence in the environment, such as tracks, tree markings, signs of ground rooting, crop damage or traffic accidents. The presence of wild boar defines the availability of a susceptible host for ASF and is thus a prerequisite for ASFV infection in wild boar, whereby modulation of the numbers of wild boar must be expected to influence disease occurrence. Despite the implied relevance of wild boar density for ASF, it is inherently difficult to determine the numbers of this adaptable wildlife species accurately and to monitor it continuously. It is, therefore, necessary, however, to estimate wild boar density values based on hunted wild boar counts per area [47], to use wild boar habitat models [41,42] or other techniques, such as photo-trapping or genetic capture–recapture approaches [48]. It is, therefore, important to keep in mind that wild boar density estimates are likely crude approximations of the true absolute value and likely biased by the hunting effort in accordance with how much a particular density estimate relies on underlying hunting bag data.

During the course of the current ASF epidemic in the European Union, the association of wild boar density with the occurrence of ASF in wild boar was studied in Estonia, Latvia, Lithuania and Poland, considering different time segments of the epidemic. Classification tree modelling of potential factors for ASF detection in wild boar from 2014 to 2016 in the Baltic States identified wild boar density as a risk factor for Latvia and Estonia, but not for Lithuania during this period [39]. These findings were confirmed by other studies that considered data during the same time segment for Estonia and for Lithuania from 2014 to 2017 [37,47]. Follow-up investigation of wild boar density in Estonia during 2014 to 2017 found, by using a generalised additive model and a Bayesian hierarchical approach, that an increase in the estimated wild boar density by one wild boar per square kilometre increased the odds of detecting ASF in wild boar by over two units [19]. This analysis was repeated with the Bayesian model, this time including data from an additional year on ASF in wild boar in Estonia, i.e., from 2014 to 2018, with the same result [9]. Another follow-up repeat of the same analysis now included Estonian data up to 2019 [22]. This analysis, however, did not find a significant association of wild boar density with the occurrence of ASF in wild boar [22]. This observation suggests that the effect of risk factors in this context could be time-dependent and that the underlying stage (e.g., early or late) of the examined ASF epidemic may influence whether a risk factor plays a role or not. A similar observation was made in Poland from 2014 to 2015 when a correlation between wild boar numbers and ASF cases in wild boar was detected for the affected forest regions only during the first year of the observations [35]. In the following year, this correlation was lost, as the ASF-affected area in Poland had further increased [35]. Another study examined the effect of wild boar density in the ASF-affected area in East Poland from 2014 to 2016 by using a generalised linear mixed model and found that wild boar density was a significant factor in predicting the occurrence of ASF in wild boar [36].

Taken together, high wild boar density appears to be associated with increased odds of ASF occurrence in wild boar in Europe. However, this observation might be time-dependent and influenced by the local stage of the ASF epidemic.

## 7. ASF Disease

Environmental factors directly related to ASF itself describe spatial and temporal patterns of the occurrence of ASF. Evidence of the occurrence of ASF in wild boar or domestic pigs in the environment of a susceptible wild boar population is likely to pose a distance-dependent risk of ASFV infection for that population. In other words, the nearness of ASF increases the probability of ASF occurrence in susceptible animals, which represents an underlying principle for animal disease control. In the European Union, the establishment of disease control zones around detected ASF outbreaks is currently implemented through Regulation (EU) 2021/605. Understanding the relevant distances that associate nearness to previous ASF cases with the increased likelihood of ASF occurrence in wild boar would help with decisions on the zoning for ASF surveillance and control measures. Knowledge about the role of spatiotemporal distance to previous ASF cases could also be used to differentiate epidemiological scenarios for possible ASFV introduction pathways into susceptible wild boar populations [9].

Temporal and spatial relationships of ASF cases in wild boar in the European Union from 2014 to 2019 were used to construct networks, in which cases represented the nodes and links between cases were assigned based on the previous occurrence in time and on minimal distance criteria [9,22]. The resulting network provided an estimation of the speed distribution of ASF spread from one case report to a neighbouring wild boar report in the network. When considering only proximity-based networks, it was found for the Baltic States and Poland during 2014 to 2018 that the median distance, with which ASF spread among wild boar during a year was 11.7 km (range of country-specific medians was 9.4–16.3) [9]. Inclusion of additional case reports up to 2019 with data from Belgium, the Czech Republic, Hungary and Romania resulted in lower ranges for the annual median spread of ASF cases in wild boar; a median ASF spread in wild boar of 5 km (the Czech Republic) to 31.4 km (Romania) per year. The 75 percentiles of the annual spread distance distributions for these countries revealed more extreme ranges of 11.7 km (the Czech Republic) to 120.3 km (Romania) [22]. Similar spread distances for ASF in wild boar were identified at about 18 km per year in Poland from 2014 to 2015 [21]. These types of analyses illustrate the distribution of distances at which susceptible wild boars may be at an increased risk of ASFV infection due to the nearness of ASF cases in their environment within a year.

The influence of the distance to previous ASF cases and the distance to the border with Belarus (as a proxy for an unknown ASF disease status) were compared for carcasses of ASFV-infected and non-infected wild boar examined during 2014 to 2016 in the outbreak area in East Poland [36]. Inclusion of the distance variables in a generalised linear mixed model revealed their significant contribution to predicting ASF occurrence in wild boar. It was found that the probability of detecting ASFV-positive wild boar carcasses increased by 19% when distances from previous cases decreased from 64 to 7 km [36]. A similar observation was made when the distance from the border with Belarus decreased from 34 to 0.07 km. Then, the probability of detecting ASF in wild boar increased by 20% [36]. Proximity to the border of another country as a substitute for ASF nearness and proximity to previously reported ASF cases were also identified as significant factors in predicting wild boar ASF occurrence in a spatial zero-inflated Poisson modelling study of ASF-positive wild boar carcass occurrence in South Korea from 2019 to 2020 [49].

The concept of ASFV transmission cycles in domestic pigs, which may involve ticks (pig–tick cycle) [50,51], or ASFV-contaminated pork products (domestic cycle), suggests that wild boar may also become infected through similar pathways in areas where ASF occurs in domestic pigs [28,29,52].

This section emphasises the nearness of ASF in the environment as an important risk factor for the occurrence of ASF in wild boar. Specifically, distances of up to about 30 km to the nearest ASF case appear to increase the probability for the occurrence of most ASF cases in wild boar during one year of exposure.

## 8. Discussion

The information reviewed herein summarises current knowledge on potential environmental risk factors that may influence the occurrence of ASF in European wild boar (Table 1). Exposure of wild boar to environmental risk factors related to climate, land cover, human activity, wild boar and ASF nearness were found to contribute to ASFV infection in this pig species (Figure 3).

The fact that ASF in wild boar dominates the current situation in Europe, especially since 2014, and the ongoing spread of ASF in this species, focuses much attention on controlling the disease in wild boar. Therefore, a comprehensive understanding of environmental risk factors for ASF in wild boar is urgently needed and essential for successful disease management. In this regard, the information gathered for this article is judged overall to be mostly of recent origin, sometimes scarce and partially inconsistent. Importantly, most of the available studies that reported information on environmental ASF risk factors and met the criteria to be selected for the review of evidence-based risk information associated with ASF occurrence in wild boar were reported from the Baltic States and Poland. This may at least in part be due to the emerging situation of ASF in wild boar in Europe. Conclusions drawn from the available data principally apply to similar geo-climatic regions. However, extrapolations to other areas of Europe, for which no comparable data exist, should be made with care. Naturally, environmental risk factors for ASF in wild boar are part of a greater ecosystem and are expected to be interlinked and interdependent. This context implies that unifactorial analysis of potential ASF risk factor effects would likely overlook relevant factors among confounding variables. Thus, multifactorial approaches were mostly employed in the reviewed studies to model the association of ASF occurrence in wild boar with the examined variables [9,16,19,22,36,39,44,49], thereby highlighting the relevance of identified risk factors among other co-variables.

Studies that investigate environmental ASF risks would mainly rely on detailed knowledge about ASFV infections in wild boar with accurate and precise information on time and space of disease occurrence, including the same data from suitable reference scenarios with non-infected wild boar. These type of data may be difficult to obtain, but the implementation of appropriate frameworks could support the collection of important information that would otherwise be lost. An example of such a framework is the CSF/ASF wild boar surveillance database (https://surv-wildboar.eu, accessed on 1 June 2021) of the European Union, run by the German Friedrich-Loeffler-Institut on behalf of the EU Reference Laboratories for Classical and African swine fever. It is expected that similar surveillance databases exist in Europe (likely at a national level) that record positive and negative test results, as well as relevant metadata for ASF samples from wild boar.

Much of the risk information contained in this review was obtained from expert-based EFSA reports [9,19,22,39]. This information is likely to be peer-reviewed in the relevant EFSA working groups and panels but may not necessarily be published in peer-reviewed journals. In any case, concordant conclusions were reached in situations where the findings of EFSA analyses were reproduced at least in parts by similar peer-reviewed studies using alternative methodologies [36,47]. Two pathways to access data on ASF in wild boar for the analysis of environmental risk factors were apparent. Epidemiologists either used information from their home country or collaborated with colleagues from other countries. Both pathways appear fruitful and should be supported to facilitate further studies of environmental risk factors for ASF in wild boar to improve ASF control in the future.

Inconsistencies were noticed among the reviewed risk factors. Some of these were differences in seasonal peaks of ASF occurrence in wild boar among the examined countries. These observations suggest that there are country-specific and seasonal differences in the factors that influence the frequency with which wild boar are ASFV infected in the studied outbreak areas. Since most ASF cases in the Baltic States and Poland fall inside a window of 15 degrees latitude, it appears unlikely that the observed inter-country variability of seasonal ASF peaks is directly caused by spatial variation in meteorological patterns alone, but more probably through variability in other, secondary seasonal factors. Such factors could be related to virus persistence in the environment, wild boar ecology, seasonal farming activities or other seasonal human activities [9]. Another reason for the observed seasonal inconsistencies could be a shorter duration of the epidemic, consequently a lack of data over several years and differences in disease control strategies. For instance, ASF outbreaks in the Czech Republic [16] or Belgium [17] were much smaller in areal size and duration, whilst the Baltic States and Poland have managed ASF in a relatively large area for multiple years now. Therefore, periodically high levels of ASF detection in smaller outbreak scenarios could be caused by intensive search efforts and subsequent elimination of the disease as a result rather than by underlying seasonal patterns. Similar effects may also contribute locally in larger outbreak scenarios. Whilst these possibilities or other potential causes remain to be investigated, the identified differences in seasonal peaks of ASF occurrence in wild boar among the examined countries could be very useful to identify additional underlying risk factors for ASF. At least for a subset of large ASF-affected European areas over long periods, such as for the Baltic States and Poland, the general seasonal pattern of ASF detection in found-dead wild boar appears to be similar in principle, presenting with more or less pronounced peaks in winter and summer [22]. Boklund and colleagues discuss potential reasons for these seasonal peaks [9], but further study is required to understand the underlying causes for the seasonal patterns of ASF occurrence in wild boar.

Another interesting inconsistency in the association of risk factors with the occurrence of ASF in wild boar appeared to be time-dependent. EFSA risk analyses of wild boar habitat quality- and road density-related risks for disease occurrence were conducted in a sequential manner for Estonia, starting with data from 2014 to 2016 [39], included additional data that became available each year thereafter until 2019 [9,19,22]. This approach provided insights into temporal dynamics for the ability of these factors to predict the occurrence of ASF in wild boar in Estonia. It was observed that both factors, wild boar habitat quality and road density, were initially associated with increasing the odds of disease occurrence, but this association was lost as the ASF epidemic progressed in 2018 and 2019 [9,22]. These results indicate that ASF spread continuously in wild boar in Estonia to most of the examined administrative units during 2018 and 2019, ultimately affecting almost the entire country [53]. This process appeared to be independent of the regional wild boar habitat quality or road density in the later stages of the epidemic in Estonia [9,22]. This conclusion further suggests that some environmental risk factors may only be relevant during the early phases of ASF outbreaks or at the epidemic front of larger outbreak areas by modulating the progression of the epidemic wave-front through the landscape [54,55]. The ASF induced collapse of the wild boar population, which may occur as the epidemic passes through an area, could be a key reason that disengages ASF occurrence from wild boar density and related risk factors, thus stunting this type of risk factor associated with disease occurrence as the epidemic progresses over time [27,47]. It is consistent with this interpretation that the correlation of detected ASF cases in wild boar and wild boar density estimates in Polish forest units was only noticeable during the first year of the epidemic in the study area but was lost during the second year [35]. These findings imply that environmental ASF risk factors should be examined during the early stages or at the leading edge of the prevailing epidemic in the study area. It is further implied that the examination should occur on a fine spatial scale [16,56], rather than a coarse regional level, to help identify relevant factors of ASF occurrence in naive wild boar populations. All things considered, it could be hypothesised that many of the reviewed risk factors are, in fact, modulating the occurrence and distribution of wild boar in the environment, rather than occurrence of ASF under the condition that wild boar are present in an area. This could explain why the association of some risk factors with ASF is lost as the epidemic progresses over time. These temporally dependent risk factors (such as wild boar habitat quality) may identify indirect, wild boar-related, rather than disease-related factors and may warrant future ASF risk factor analyses that consider ASF occurrence under the condition of wild boar presence.

## 9. Conclusions

The current understanding of environmental ASF risk factors has implications for disease management in European wild boar and provides further insights into ASF transmission dynamics. While climate, land cover and ASF-related factors would be difficult to control, they may provide guidance on how to allocate resources. Knowledge of human activity and wild boar-related factors might offer opportunities for direct control. The reviewed information about environmental ASF risk factors in wild boar implies the following considerations for managing the disease in regards to ASF surveillance, hunting, wild life management, wild boar carcass searches, implementation of barriers to reduce wild boar migration, forestry and cropping activities [57]:

### 9.1. Timing

Adapt the timing of disease surveillance and control efforts to the seasonal ASF occurrence pattern in wild boar. Increasing active wild boar carcass search and removal efforts during times of peak disease occurrence, such as during summer or winter, may improve the efficiency of removing carcasses of ASFV-infected wild boar from the environment. However, constraints due to extreme weather or vegetation may impede such seasonal efforts but could also be advantageous. During warm temperatures in summer, it may be more likely to find carcasses of ASFV-infected wild boar closer to water sources, thus spatially restricting the targeted search area in summer. Seasonally focussed search efforts may release resources during other times of the year, such as spring and autumn, which could then be used for other control activities, such as regulatory work. Seasonal considerations may further be adapted to known seasonal wild boar behaviour patterns and the regulation of agricultural cropping activities in the area, as cropping activities likely influence land cover, wild boar habitat suitability and potentially also occurrence patterns of ASF. Likewise, active surveillance may be improved by well-planned hunting of wild boar during late summer and autumn, particularly in areas currently not affected by ASF and which are adjacent to outbreak regions, all whilst following suitable biosecurity measures. This strategy may increase the chance of detecting the recently infected live animals at the epidemic ASF front.

### 9.2. Spatial Targeting

Adapt the spatial targeting of disease surveillance and control efforts to the spatial pattern of ASF occurrence in wild boar by prioritising areas to which many spatially informative environmental ASF risk factors apply. High priority areas for wild boar carcass searches may be forested areas, particularly those with younger tree stands, as well as forests with large proportions of broad-leafed trees, areas that are no more than 500 m away from the edge of the forest and from water sources, whilst potentially focussing on areas over 100 m away from roads. In open meadows, tall herb layer areas could be prioritised, again less than 500 m from water. These land cover factors may identify areas of high habitat suitability for wild boar and thus high wild boar densities. Since associations of land cover- and wild boar-related factors with the detection of ASF in wild boar were lost over time, prioritisation of these high-risk areas may be particularly important in the early phases of an outbreak or at the edges of established outbreak areas. Similar spatial considerations could be applied for surveillance purposes to target the hunting of wild boar adjacent to ASF outbreak areas, but also to position fences for ASF control or to establish wild boar culling zones (‘white zones’). By mapping high-risk areas of ASF occurrence in wild boar according to the identified criteria, it may be possible to identify landscape corridors of high and low disease risk. Bosch et al. previously proposed a similar concept to harness knowledge about areas of high wild boar habitat suitability for the management of ASF [41]. The characterisation of disease risk areas on a small spatial scale may allow locating fences and ‘white zones’ along low-risk corridors to potentially enhance their barrier function. High-risk corridors, on the other hand, would represent anticipated pathways of ASF spread and can be targeted with appropriate measures to prevent ASF spread.

### 9.3. Dynamic Disease Control

The spatial association of known and newly detected ASF cases over time implies continuous spatial disease spread. This may therefore indicate that the size of ASF restriction zones, which define disease control activities, need to be continuously increased as well. A concept of dynamically growing ASF restriction zones over time and in relation to known disease occurrence could improve the spatial overlap of the ASF affected area and the applied control measures, thus avoiding that disease control lags spatially behind ASF occurrence. It is important to note, however, that a system of continuously expanding restriction zones needs to be effectively managed and would require the appropriate allocation of limited resources. Moreover, the expectance of ASF spread in wild boar is based on the analysis of spatially extended disease events in the Baltic States and Poland that may not apply to other ASF-affected areas in Europe. Therefore, a model of disease-anticipating, dynamic expansion of ASF restriction zones may not only be undesirable but also not justified in a specific local context.

In summary, the presence of ASF in European wild boar has a devastating impact on the European pig industry and causes significant hardship for people that work with or are dependent on pigs and pig products. The awareness of potential environmental ASF risk factors is critical in this situation, as ASF is very difficult to control in wild boar. Due to their inherent nature, the association of many environmental factors with ASF is challenging to study, and as a result, evidence is scarce. Nevertheless, at least some aspects of ASF occurrence in wild boar appear to be far from random, and environmental risk factors conducive to the observed disease pattern remain to be determined through careful epidemiological study. Notably, unpredictable, human-mediated spread events of ASF in wild boar occur, and reasonable allowances for these events have to be made for the investigation of environmental ASF risk factors.

The conclusions that can be derived from the current knowledge about environmental ASF risk factors for European wild boar should be applied to disease management. In the meantime, and particularly if ASF has been reported nearby, it would be fair to exercise extreme caution in relation to human activities that directly or indirectly interact with wild boar in the widest sense. These activities include domestic pig husbandry, waste handling, wildlife management, hunting practices, forestry work, recreational outdoor movements or any similar type activities for which adequate control and biosecurity measures should be carefully implemented.

## Figures and Tables

**Table 1:** Summary of potential environmental risk factors for the occurrence of African swine fever in wild boar.

Risk Factor	Summary of the Possible Effect	Reference
Seasonality	Seasonal disease patterns of disease occurrence observed	Podgorski et al., 2018 [21]; Smietanka et al., 2016 [35]; Podgorski et al., 2020 [36]; Pautienius et al., 2018 [37]; Maciulskis et al., 2020 [38]; EFSA, 2018 [9]; EFSA (Abrahantes et al.), 2017 [39]; EFSA, 2020 [22]; EFSA (Depner et al.), 2017 [19]
Precipitation	Precipitation during extreme dry, wet or cold periods influences disease occurrence	Liang et al., 2020 [40]
Temperature	Temperatures, particularly during extremely cold periods, may influence disease occurrence and spatial association with water sources	Liang et al., 2020 [40]; EFSA, 2018 [9]; EFSA, 2020 [22]; Cukor et al., 2020 [16]
Forest	More forest, proximity to forest, younger tree ages of broad-leafed forest associated with disease	EFSA (Abrahantes et al.), 2017 [39]; Loi et al., 2019 [44]; Podgorski et al., 2020 [36]; Cukor et al., 2020 [16]
Water	Presence and proximity to surface water associated with disease	EFSA (Abrahantes et al.), 2017 [39]; Cukor et al., 2020 [16]
Meadows	Growth height of meadow vegetation between 1 and 1.2 m associated with disease detection	Cukor et al., 2020 [16]
Wild boar habitat quality	High wild boar habitat suitability likely associated with disease	EFSA, 2018 [9]; EFSA (Depner et al.), 2017 [19]; EFSA, 2020 [22]
Human population density	Greater human population density may be associated with disease	EFSA (Abrahantes et al.), 2017 [39]
Human settlements	Human settlements unlikely associated with disease	EFSA (Abrahantes et al.), 2017 [39]; EFSA (Depner et al.), 2017 [19]; EFSA, 2018 [9]; EFSA, 2020 [22]; Podgorski et al., 2020 [36]; Cukor et al., 2020 [16]
Roads	More roads may increase detection of disease, but roads may also have a dispersing effect on disease occurrence	EFSA (Abrahantes et al.), 2017 [39]; Podgorski et al., 2020 [36]; EFSA (Depner et al.), 2017 [19]; EFSA, 2018 [9]; EFSA, 2020 [22]; Cukor et al., 2020 [16]; Schulz et al., 2020 [46]
Renewable energy production	More energy production from renewable resources may reduce disease	Loi et al., 2019 [44]
Waste production	More waste production likely associated with disease	Loi et al., 2019 [44]
Hunting	Hunting was not found to associate with disease	EFSA, 2018 [9]; EFSA, 2020 [22]
Farming	More domestic pigs and pig farms, particularly smaller pig farms, associated with disease occurrence	EFSA (Abrahantes et al.), 2017 [39]; EFSA (Depner et al.), 2017 [19]; EFSA, 2018 [9]; EFSA, 2020 [22]
Wild boar presence	High wild boar density associated with disease	EFSA (Abrahantes et al.), 2017 [39]; Pautienius et al., 2018 [37]; Nurmoja et al., 2017 [47]; EFSA (Depner et al.), 2017 [19]; EFSA, 2018 [9]; EFSA, 2020 [22]; Smietanka et al., 2016 [35]; Podgorski et al., 2020 [36]
ASF nearness in wild boar	Proximity to ASF in wild boar associated with disease	EFSA, 2018 [9]; EFSA, 2020 [22]; Podgorski et al., 2018 [21]; Podgorski et al., 2020 [36]; Lim et al., 2021 [49]

## Data Availability

Data from the OIE is publicly available under (https://wahis.oie.int/#/home, accessed on 1 June 2021). The underlying data from ADIS is not public and only available to the competent authorities responsible for animal health in countries providing data on outbreaks of selected contagious animal diseases and to the European Commission services. Tabulated and cartographical summaries of current outbreak information collected by ADIS is provided by the EU (https://ec.europa.eu/food/animals/animal-diseases/animal-disease-information-system-adis_en, accessed on 1 June 2021). Summaries of used data can be provided upon reasonable request to the authors.
